# Addition of docetaxel or bisphosphonates to standard of care in men with localised or metastatic, hormone-sensitive prostate cancer: a systematic review and meta-analyses of aggregate data

**DOI:** 10.1016/S1470-2045(15)00489-1

**Published:** 2016-02

**Authors:** Claire L Vale, Sarah Burdett, Larysa H M Rydzewska, Laurence Albiges, Noel W Clarke, David Fisher, Karim Fizazi, Gwenaelle Gravis, Nicholas D James, Malcolm D Mason, Mahesh K B Parmar, Christopher J Sweeney, Matthew R Sydes, Bertrand Tombal, Jayne F Tierney

**Affiliations:** a MRC Clinical Trials Unit at UCL, London, UK; b Institut Gustave Roussy, Paris, France; c Department of Urology, The Christie and Salford Royal NHS Foundation Trusts, Manchester, UK; d Department of Medical Oncology, Institut Paoli Calmettes, Marseille, France; e Warwick Cancer Research Unit, University of Warwick, Coventry, UK; f University Hospitals Birmingham NHS Foundation Trust, The Medical School, University of Birmingham, Birmingham, UK; g Cardiff University School of Medicine, Velindre Hospital, Cardiff, UK; h Lank Center for Genitourinary Cancer, Dana-Farber Cancer Institute, Boston, MA, USA; i Cliniques Universitaires St Luc, Université Catholique de Louvain, Brussels, Belgium

## Abstract

**Background:**

Results from large randomised controlled trials combining docetaxel or bisphosphonates with standard of care in hormone-sensitive prostate cancer have emerged. In order to investigate the effects of these therapies and to respond to emerging evidence, we aimed to systematically review all relevant trials using a framework for adaptive meta-analysis.

**Methods:**

For this systematic review and meta-analysis, we searched MEDLINE, Embase, LILACS, and the Cochrane Central Register of Controlled Trials, trial registers, conference proceedings, review articles, and reference lists of trial publications for all relevant randomised controlled trials (published, unpublished, and ongoing) comparing either standard of care with or without docetaxel or standard of care with or without bisphosphonates for men with high-risk localised or metastatic hormone-sensitive prostate cancer. For each trial, we extracted hazard ratios (HRs) of the effects of docetaxel or bisphosphonates on survival (time from randomisation until death from any cause) and failure-free survival (time from randomisation to biochemical or clinical failure or death from any cause) from published trial reports or presentations or obtained them directly from trial investigators. HRs were combined using the fixed-effect model (Mantel-Haenzsel).

**Findings:**

We identified five eligible randomised controlled trials of docetaxel in men with metastatic (M1) disease. Results from three (CHAARTED, GETUG-15, STAMPEDE) of these trials (2992 [93%] of 3206 men randomised) showed that the addition of docetaxel to standard of care improved survival. The HR of 0·77 (95% CI 0·68–0·87; p<0·0001) translates to an absolute improvement in 4-year survival of 9% (95% CI 5–14). Docetaxel in addition to standard of care also improved failure-free survival, with the HR of 0·64 (0·58–0·70; p<0·0001) translating into a reduction in absolute 4-year failure rates of 16% (95% CI 12–19). We identified 11 trials of docetaxel for men with locally advanced disease (M0). Survival results from three (GETUG-12, RTOG 0521, STAMPEDE) of these trials (2121 [53%] of 3978 men) showed no evidence of a benefit from the addition of docetaxel (HR 0·87 [95% CI 0·69–1·09]; p=0·218), whereas failure-free survival data from four (GETUG-12, RTOG 0521, STAMPEDE, TAX 3501) of these trials (2348 [59%] of 3978 men) showed that docetaxel improved failure-free survival (0·70 [0·61–0·81]; p<0·0001), which translates into a reduced absolute 4-year failure rate of 8% (5–10). We identified seven eligible randomised controlled trials of bisphosphonates for men with M1 disease. Survival results from three of these trials (2740 [88%] of 3109 men) showed that addition of bisphosphonates improved survival (0·88 [0·79–0·98]; p=0·025), which translates to 5% (1–8) absolute improvement, but this result was influenced by the positive result of one trial of sodium clodronate, and we found no evidence of a benefit from the addition of zoledronic acid (0·94 [0·83–1·07]; p=0·323), which translates to an absolute improvement in survival of 2% (−3 to 7). Of 17 trials of bisphosphonates for men with M0 disease, survival results from four trials (4079 [66%] of 6220 men) showed no evidence of benefit from the addition of bisphosphonates (1·03 [0·89–1·18]; p=0·724) or zoledronic acid (0·98 [0·82–1·16]; p=0·782). Failure-free survival definitions were too inconsistent for formal meta-analyses for the bisphosphonate trials.

**Interpretation:**

The addition of docetaxel to standard of care should be considered standard care for men with M1 hormone-sensitive prostate cancer who are starting treatment for the first time. More evidence on the effects of docetaxel on survival is needed in the M0 disease setting. No evidence exists to suggest that zoledronic acid improves survival in men with M1 or M0 disease, and any potential benefit is probably small.

**Funding:**

Medical Research Council UK.

## Introduction

Prostate cancer is a major health problem worldwide and is the second most common cancer in men. With 1·1 million diagnoses (15% of all cancers diagnosed in men) and 307 000 deaths estimated to have taken place in 2012, prostate cancer has become the fifth leading cause of death from cancer in men worldwide.^1^

For many decades, initial (first-line) treatments for both locally advanced and metastatic prostate cancer have been surgical castration by bilateral orchidectomy or androgen deprivation therapy with luteinising hormone-releasing hormone agonists or antagonists.^2^ The aim of these approaches is to reduce testosterone concentrations. However, the disease progresses in virtually all patients who have metastatic disease and in many patients with non-metastatic disease.^3^, ^4^ A number of treatments, such as bisphosphonates, cytotoxic chemotherapy, new hormone therapies, and radium-223, have therefore been assessed in combination with primary androgen deprivation therapy with the aim of reducing progression rates and improving survival.

One such treatment, docetaxel (given with or without estramustine), was shown in two pivotal randomised controlled trials^5^, ^6^ to improve survival in men with castrate-resistant prostate cancer that was no longer responsive to testosterone suppression alone. This finding led to the international approval by regulatory authorities of docetaxel for this disease setting and a number of randomised controlled trials, in which men with metastatic or high-risk localised prostate cancer, starting long-term androgen deprivation therapy for the first time, were randomly assigned to receive standard androgen deprivation therapy-based treatment alone or supplemented with docetaxel (with or without other agents). Results from some of the largest of these trials have now emerged. In the CHAARTED^7^ and STAMPEDE^8^ trials, men with metastatic disease had significant improvements in survival with the addition of docetaxel, whereas results of the similar GETUG-15 trial^9^, ^10^ showed no evidence of a survival benefit from docetaxel. A small number of trials of docetaxel for men with non-metastatic disease have produced promising results for relapse or failure-free survival, but the effect on survival is unclear.

Bisphosphonates are a class of drugs that have been shown to have a number of anti-cancer effects.^11^ In randomised controlled trials, the first-generation bisphosphonate, clodronate, delayed time to progression in men with bone metastases when given alongside long-term androgen deprivation therapy. Some evidence suggests that biphosphonates might improve survival.^12^ Newer (third-generation) bisphosphonates, notably zoledronic acid, have been found to reduce the risk of skeletal complications (eg, fractures) in patients with bone metastases from breast cancer and castrate-resistant prostate cancer.^13^ In the wake of these results, a number of randomised controlled trials have been designed to investigate whether men who are commencing long-term androgen deprivation therapy for either metastatic or localised hormone-sensitive prostate cancer benefit from bisphosphonates.

As part of the wider Systemic Treatment Options for Prostate Cancer (STOpCaP) meta-analysis project, we aimed to systematically review all relevant randomised controlled trials that tested the addition of docetaxel or bisphosphonates to standard of care. We prospectively planned meta-analyses that would respond and adapt to the emergence of new trial results, while also assessing the potential effect of trials that are yet to be completed or reported.

## Methods

### Systematic review and framework for adaptive meta-analysis

Standard systematic reviews of both aggregate and individual participant data can take many years to complete and are usually retrospective, so they cannot always keep pace with therapeutic developments. We therefore used a framework for adaptive meta-analysis (FAME) being developed by the MRC Clinical Trials Unit at UCL (London, UK) to rapidly and robustly assess the effects of therapies and to respond to emerging evidence. The key principle is to systematically identify all trials using established methods, then synthesise what is already known about the effects of therapies from aggregate data, and consider how trials that are ongoing or yet to be reported might affect these results. Thus, we deliberately began the review process before many trials of docetaxel and bisphosphonates had been completed and reported so as to build a picture of how information and evidence of the effects of these drugs might accumulate. This review process allowed us to decide prospectively when we were likely to have sufficient results or power, or both, for reliable aggregate data meta-analyses and to interpret our results, taking into account the possible effect of any as yet unavailable evidence. This also helped us determine the potential value of updating meta-analyses, and whether these meta-analyses should be based on aggregate data or individual patient data.

### Study selection and data extraction

Randomised controlled trials comparing either standard of care versus standard of care plus docetaxel or standard of care versus standard of care plus bisphosphonate (at a therapeutic dose) were eligible if they aimed to include men with high-risk localised or metastatic, hormone-sensitive (ie, not castrate-resistant) prostate cancer. We had no formal exclusion criteria.

We sought to identify all trials, irrespective of whether a trial was ongoing or completed, published or unpublished, with no language restrictions. We searched MEDLINE,^14^ Embase,^15^ LILACS,^16^ and the Cochrane Central Register of Controlled Trials from inception to Sept 30, 2015, using filters to include only randomised controlled trials. These searches were supplemented by searching trial registers, conference proceedings, review articles, and reference lists of trial publications (appendix pp 1–5). Collaborators were asked throughout the project if they knew of any additional trials. CLV, LHMR, and SB assessed all relevant trial reports or protocols. Search terms used are listed in the appendix.

For all eligible trials, we extracted data on: the accrual period, actual or (if ongoing) planned number of participants; whether previous androgen deprivation therapy was allowed; control group treatments (eg, type of androgen deprivation therapy used); docetaxel dose and scheduling; bisphosphonate type; dose and duration of bisphosphonate treatment; median patient age; metastatic status; performance status; TNM status; Gleason score; and median PSA concentration at the start of androgen deprivation therapy. We also extracted reported survival and failure-free survival results by trial and by participant subgroup (if available) from published reports and presentations. If insufficient data were available from published reports, we sought it directly from study investigators. We also extracted data on methods of sequence generation, allocation concealment, completeness of outcome data reporting, and attrition from trial reports or protocols, or both, to assess the risk of bias of individual trials.^17^

Methods were prespecified and are available in an online protocol.

### Outcomes

The primary outcome, survival, was defined as the time from randomisation until death from any cause. The secondary outcome was failure-free survival. Although there is no widely accepted definition of failure-free survival, for the purpose of this systematic review and meta-analysis, we defined it as the time from randomisation to biochemical failure, clinical failure (local relapse or metastases), or death from any cause.

### Statistical analysis

From our review of the completed and ongoing trials, we anticipated that results from the three largest trials of docetaxel in M1 disease, which included around 90% of all potential participants, would become available by June, 2015, with a median follow-up of about 3–4 years. The typical 4-year survival reported in trials in this group of men was 40%, which we set as our baseline for predicting the power a meta-analysis of these trials would be likely to provide. We estimated that we would have about 70% power to detect an absolute difference of 5% in 4-year survival (hazard ratio [HR] 0·87) and more than 99% power to detect a 10% difference in 4-year survival (HR 0·75); these are the sort of moderate effects one might expect in advanced prostate cancer. For the bisphosphonate comparison in M1 disease, we predicted that we would have results from trials that included about 85% of all potential participants and, using the same baseline survival, would achieve about 65% power to detect an absolute difference of 5% in 4-year survival and more than 99% power to detect a 10% difference in 4-year survival. These estimates gave us a clear trigger to conduct meta-analyses in the M1 disease setting.

We were aware that mature results of trials in M0 disease would lag behind those in the M1 setting owing to a more favourable prognosis, so we expected fewer data for the docetaxel and bisphosphonate comparisons (around 60% of potential participants). Nevertheless, on the basis of an average baseline 4-year survival of around 80% in the reported trials, we predicted that we would still have reasonable power (60%) to detect a 5% difference in 4-year survival and more than 99% power to detect a 10% difference in 4-year survival, allowing us to compare the evidence between the two settings and ascertain if and when further meta-analyses are needed.

For each trial, we extracted HRs of the effects of docetaxel or bisphosphonates on survival and failure-free survival from trial reports, estimated them from published Kaplan-Meier curves or other summary statistics,^18^, ^19^, ^20^ or obtained them directly from trialists. For those multiarm trials^8^, ^21^ for which HRs were not available for the comparison of interest, we obtained these data indirectly from other HRs. For example, we could obtain the HR for the addition of docetaxel to standard of care plus zoledronic acid versus standard of care plus zoledronic acid alone for the STAMPEDE trial^8^ from the ratio of the HRs for the separate comparisons of standard of care with or without zoledronic acid and standard of care with or without zoledronic acid plus docetaxel.

We combined the HRs from each of the individual, eligible trials in a meta-analysis using the fixed-effect model (Mantel-Haenzsel). We also used the random-effects model to assess the robustness of the results to the choice of this model for the primary analysis.^22^

We assessed the heterogeneity in treatment effects between trials using the *I*^2^ statistic and χ^2^ test. We planned to combine all trials and, providing that sufficient trials or data were available, preplanned analyses that would compare trials (or patients within trials) grouped by metastatic status, use of previous local treatment for prostate cancer, planned radiotherapy as part of the standard of care, type of and length of time on androgen deprivation therapy allowed before randomisation, total planned dose of docetaxel, additional agents in the docetaxel group only, type of bisphosphonate, and dose of zoledronic acid. We aimed to calculate a meta-analysis HR for each group and test for differences between the groups using a χ^2^ test for interaction and *F* ratio.^23^ If we found differences in effect by metastatic status, we planned to carry out the other trial group analyses separately within the M1 and M0 groups. We also planned to investigate whether there were interactions between any treatment effect and any of the following covariates: age; performance status; TNM stage; Gleason score; whether newly diagnosed or not; previous androgen deprivation therapy; and (for M1 disease only) the location and volume of all metastases and the volume of bone metastases. The interaction HR in each trial was calculated from the ratio of the estimated HRs for each subgroup (eg, the HR for previous androgen deprivation therapy divided by the HR for no previous androgen deprivation therapy); these HRs were then combined across trials using a fixed-effect meta-analysis.^24^ We used Stata version 13 for all analyses.

### Role of the funding source

The funder of the study had no role in study design, data collection, data analysis, data interpretation, or writing of the report. The corresponding author had full access to all the data in the study and had final responsibility for the decision to submit for publication.

## Results

**Figure 1:**
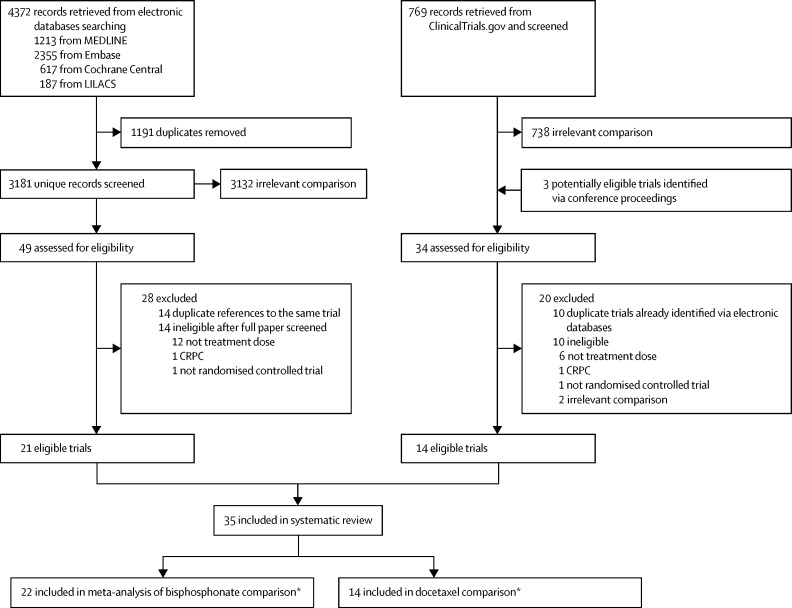
Study flow chart CRPC=castrate-resistant prostate cancer. *One trial (STAMPEDE)^8^ is eligible to be included in both docetaxel and bisphosphonate comparisons.

Our searches of bibliographic databases, trial registers, and conference proceedings identified 5141 articles and records (figure 1). After removing obvious duplicates and records that were clearly irrelevant, 83 records of potentially eligible trials were thoroughly scrutinised. 24 of these records were duplicates, and a further 24 records were ineligible. In total, 35 trials were eligible; 14 trials were eligible for inclusion in the docetaxel comparison, and 22 trials were eligible for the bisphosphonate comparison (table 1, table 2).^25^, ^26^, ^27^, ^28^, ^29^, ^30^, ^31^, ^32^, ^33^, ^34^, ^35^, ^36^, ^37^, ^38^, ^39^, ^40^, ^41^, ^42^, ^43^, ^44^, ^45^, ^46^, ^47^ One large multiarm trial (STAMPEDE),^8^ which incorporates multiple treatment comparisons in men with both M0 and M1 disease, contributes to both the docetaxel and bisphosphonates meta-analyses.

Five trials compared standard of care with or without docetaxel in men with M1 disease. One trial (GOUP 01/04 [NCT00796458]), including 200 men, is still recruiting, and another trial,^32^ including 14 men, has yet to report suitable outcome data (table 2). In the three remaining trials,^7^, ^8^, ^10^ men aged 36–91 years (median 63–66 years) with a good performance status received either androgen deprivation therapy-based treatments (standard of care) with or without docetaxel (table 1). Most men had presented with metastatic disease and were starting long-term androgen deprivation therapy for the first time. Docetaxel was given at a standard dose of 75 mg/m^2^ per cycle every 3 weeks for six to nine cycles, and median follow-up ranged from 29 months to 82·9 months (table 1). All trials were assessed as being at low risk of bias (table 3).

**Figure 2:**
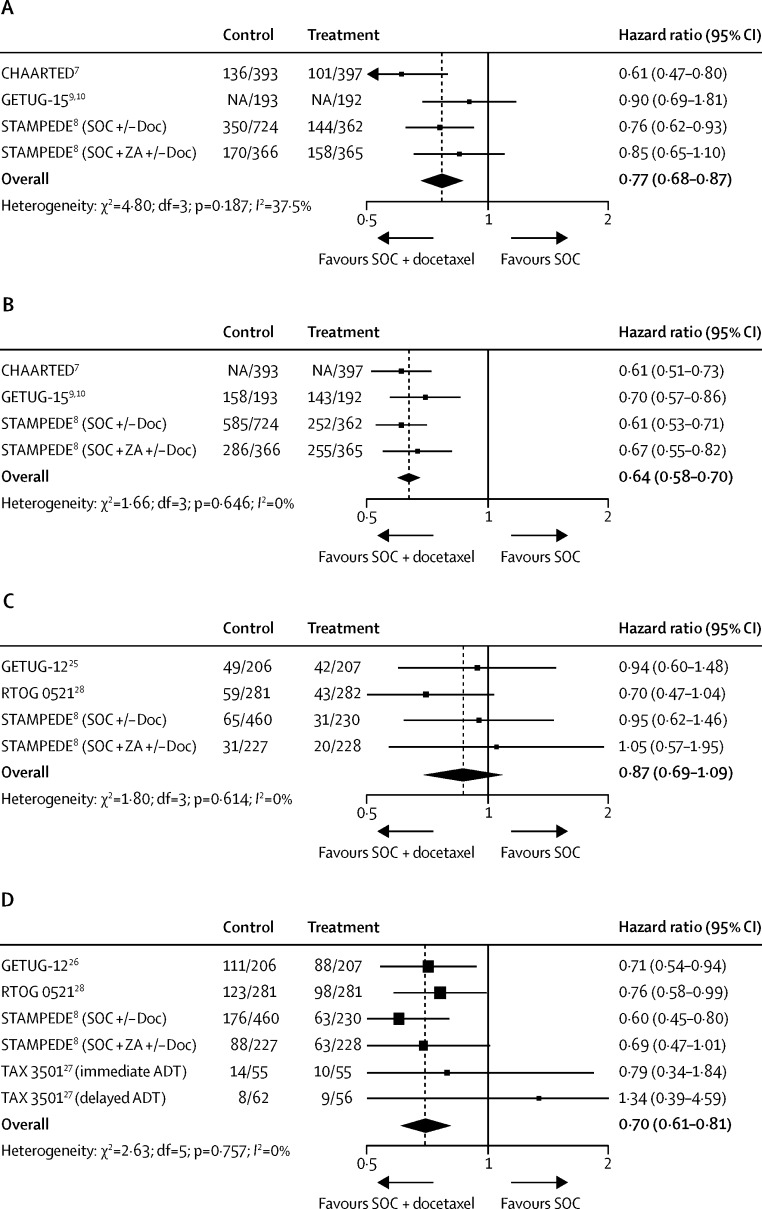
Effect of addition of docetaxel to standard of care on survival and failure-free survival (A) Effect of the addition of docetaxel on survival in men with M1 disease. (B) Effect of the addition of docetaxel on failure-free survival in men with M1 disease. (C) Effect of the addition of docetaxel on survival in men with M0 disease. (D) Effect of the addition of docetaxel on failure-free survival in men with M0 disease. NA=event numbers by group not available. SOC=standard of care.

Survival data from these three trials^7^, ^8^, ^10^ were available for 2992 (93%) of 3206 men with M1 disease (table 1), and 1271 deaths had been recorded. Assuming a typical 4-year survival with standard of care of 40%, the meta-analysis HR of 0·77 (95% CI 0·68–0·87; p<0·0001), translates to a 9% (95% CI 5–14) absolute improvement with standard of care plus docetaxel relative to standard of care alone (figure 2). We found no evidence of variation between the trial results. Statistical heterogeneity was very low throughout all analyses, so the estimates generated using a random-effects model were consistent with those generated with the fixed-effect model.

Failure-free survival was defined similarly in all trials. However, in the STAMPEDE trial,^8^ only prostate cancer specific deaths were included (rather than death by any cause), and in the CHAARTED trial^7^ the most similar reported outcome to our definition of failure-free survival was time to hormone-refractory disease, which was defined as the time from randomisation until clinical or serological progression. Results were available for the same 2992 men as for survival, and 2204 events were recorded. Assuming a baseline 4-year failure-free survival of 20%, the meta-analysis HR of 0·64 (95% CI 0·58–0·70; p<0·0001) translates to a 16% (95% CI 12–19) improvement, reducing failures from 80% to 64% (figure 2). Again, we found no evidence of variation between the trial results.

We identified 11 trials that compared standard of care with or without docetaxel for men with non-metastatic disease (M0). Two trials (CAN-NCIC-PR12 [NCT00651326] and 05-043 [NCT00116142]), including 398 men, have finished accrual but have yet to report any results. Five trials,^31^, ^32^, ^33^, ^34^, ^35^ including 1196 men, have yet to report any survival outcomes (table 2). The four remaining trials,^8^, ^25^, ^27^, ^28^ all of which have reported survival or failure-free survival, or both, were included in the meta-analysis. Men of median age 62–66 years (ranges not reported for all trials) with non-metastatic disease and good performance status were randomly assigned to receive standard of care with or without docetaxel (table 1). Docetaxel was given at a standard dose of 75 mg/m^2^ per cycle every 3 weeks for six cycles, except in one trial,^25^ which used docetaxel 70 mg/m^2^ plus estramustine 10 mg/kg on days 1–5 of each cycle. Median follow-up across the trials ranged from 39 months to 90 months (table 1). All trials were assessed as being at low risk of bias (table 3).

Survival data were available for 2121 (53%) of 3978 men from three of the four trials,^8^, ^25^, ^28^ and 340 deaths have been recorded. The meta-analys is HR of 0·87 (95% CI 0·69–1·09; p=0·218) translates to a potential absolute improvement of 2% (95% CI −2 to 7), assuming a typical baseline 4-year survival of 80% (figure 2); however, the confidence intervals are wide, and the result is not statistically significant. We found no evidence of variation between the trial results.

Failure-free survival was defined consistently in all four trials,^8^, ^25^, ^27^, ^28^ but in the STAMPEDE trial,^8^ only prostate cancer-specific deaths were included (rather than death by any cause), and the GETUG-12 trial^26^ included time-to-salvage treatment. Results were available for 2348 (59%) of the 3798 men included in all four trials, and 851 events have been recorded. The meta-analysis HR of 0·70 (95% CI 0·61–0·81; p<0·0001) translates to an absolute improvement of 8% (95% CI 5–10), reducing 4-year failure rates from 30% to 22%, assuming a baseline 4-year failure-free survival of 70% (figure 2). Again, no evidence exists of variation between the trial results.

We identified seven trials that compared standard of care with or without bisphosphonates in men with M1 disease. The results of one trial (KYUH-TRIGU0705 [NCT00685646]), including 227 men, have yet to be reported, and in three other trials,^37^, ^42^, ^47^ including 142 men, skeletal-related events, changes in bone mineral density, or both were the primary outcomes, and survival was not reported (table 2). In the three remaining trials,^8^, ^12^, ^30^ men of median age 66–71 years (range 40–88) with good performance status were randomly assigned to receive standard of care with or without either zoledronic acid^8^, ^30^ or sodium clodronate^12^ (table 1). Zoledronic acid was given at a dose of 4 mg every 3–4 weeks for either 2 years or until disease progression. Sodium clodronate was given orally at 2080 mg daily for up to 3 years (table 1). Median follow-up in the trials ranged from 24·4 months to 138 months (table 1). All trials were assessed as being at low risk of bias (table 3).

**Figure 3:**
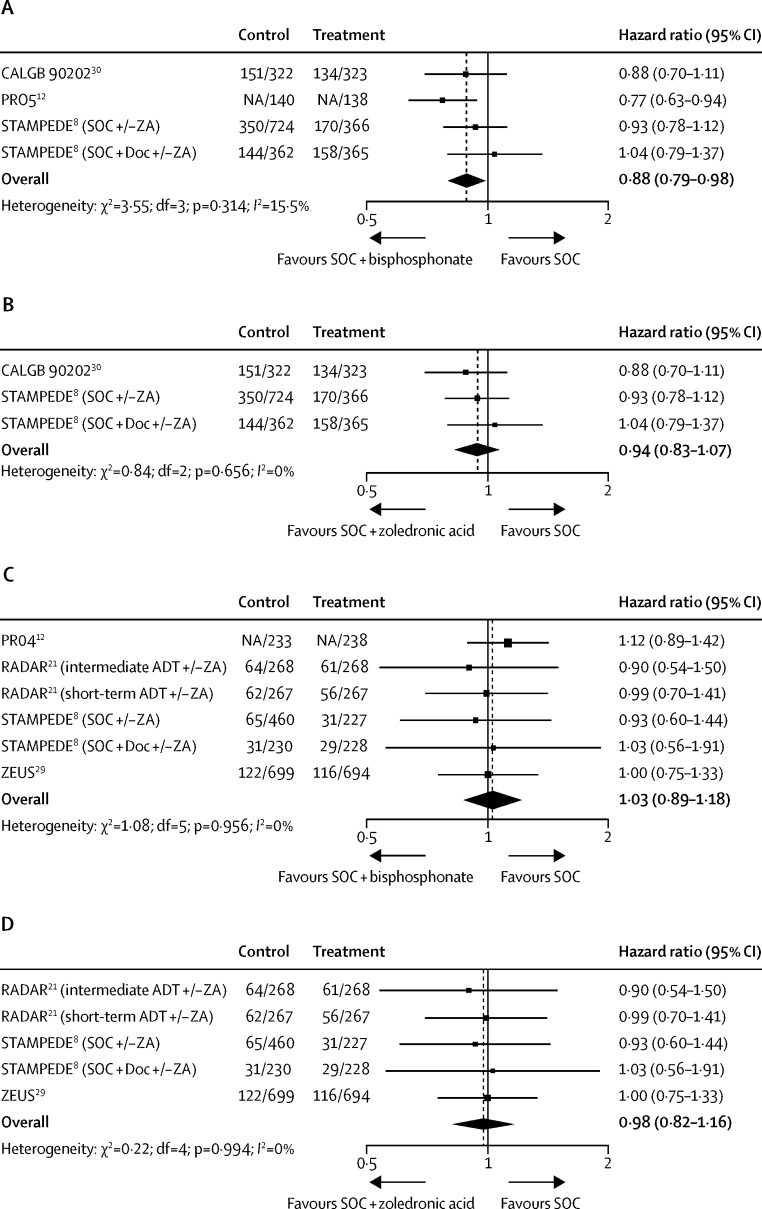
Effect of addition of bisphosphonates to standard of care on survival (A) Effect of the addition of bisphosphonates on survival in men with M1 disease. (B) Effect of the addition of zoledronic acid on survival in men with M1 disease. (C) Effect of the addition of bisphosphonates on survival in men with M0 disease. (D) Effect of the addition of zoledronic acid on survival in men with M0 disease. NA=event numbers by group not available. SOC=standard of care.

Survival results were available for 2740 (88%) of 3109 men from three trials, and 1365 deaths have been recorded. The meta-analysis HR of 0·88 (95% CI 0·79–0·98; p=0·025) translates to a 5% (95% CI 1–8) absolute improvement with standard of care plus bisphosphonates, assuming a baseline 4-year survival of 40% in men with M1 disease (figure 3). We found no evidence of variation between the trial results. However, when the analysis was restricted to the two trials (1107 deaths among 2462 men) that compared standard of care with and without zoledronic acid, we found no evidence of a benefit of standard of care plus zoledronic acid (HR 0·94 [95% CI 0·83–1·07]; p=0·323), with a potential absolute improvement in survival of 2% (95% CI −3 to 7; figure 3), although these differences were not statistically significant. In the one trial of sodium clodronate,^12^ a clear treatment benefit was reported (HR 0·77 [95% CI 0·60–0·98], p=0·032).

Failure-free survival was only reported in one trial, with other trials reporting a variety of intermediate outcomes (eg, bone metastases-free survival, time to first skeletal-related event), such that no formal meta-analysis was possible.

We identified 17 trials that compared standard of care with or without bisphosphonates for men with M0 disease. The results of three trials (CECOG [NCT00181584], ZENITH [NCT00063609], and NU-02U1 [NCT00058188]), including 646 men, are unpublished, and ten other trials,^36^, ^37^, ^38^, ^39^, ^40^, ^41^, ^43^, ^44^, ^45^, ^46^ including 1494 men, have reported results for outcomes other than survival (table 2). In the four remaining trials^8^, ^12^, ^21^, ^29^ included in the meta-analysis, men aged 40–87 years (median 66–70 years) with good performance status were randomly assigned to receive standard of care with or without either zoledronic acid^8^, ^21^, ^29^ or sodium clodronate^12^ (table 1). In two of the trials,^21^, ^29^ zoledronic acid was given at a dose of 4 mg every 3 months for either 18 months or 4 years, whereas in the third trial,^8^ zoledronic acid 4 mg was given every 3 weeks for 2 years. Sodium clodronate was given orally at 2080 mg every day for up to 5 years. Median follow-up across the trials ranged from 42 to 144 months (table 1). All trials were assessed as being at low risk of bias (table 3).

Survival results were available for 4079 (66%) of 6220 men from four trials, and 918 deaths have been recorded. We found no evidence that bisphosphonates improve survival when added to standard of care (HR 1·03 [95% CI 0·89–1·18]; p=0·724). Assuming a baseline 4-year survival of 80%, this HR translates to a potential absolute detriment in survival of 1% (95% CI −3 to 2; figure 3), although this is not statistically significant, and we found no evidence of variation between the trial results. Results were similar when the analysis was restricted to trials that tested standard of care with or without zoledronic acid (three trials, 637 deaths, 3608 men; HR 0·98 [0·82–1·16]; p=0·782), suggesting no potential absolute improvement in survival (0%, [95% CI −3 to 3]; figure 3), again with no evidence of variation between the trial results. Failure-free survival was only reported in one trial so no formal meta-analysis was done.

For both the docetaxel and bisphosphonate comparisons, far fewer results were available for the M0 disease setting than for the M1 setting, which is why the meta-analyses for the M1 and M0 settings are presented separately. Moreover, within these meta-analyses, not enough trials have assessed whether any effect varied by other trial characteristics (eg, use of radiotherapy plus androgen deprivation therapy). Also, results by patient subgroup were either too sparse, or the definitions too inconsistent, to allow for meaningful analyses from the available reported data.

## Discussion

This meta-analysis provides substantial and reliable evidence that adding docetaxel to standard of care improves the survival of men with M1 disease, with an absolute improvement of around 9% at 4 years. For men with M0 disease, evidence to date supports an 8% reduction in absolute failure rates at 4 years with docetaxel, but the evidence is insufficient to reliably assess the effects on survival. Although evidence suggests improved survival with the addition of bisphosphonates to standard of care for men with M1 prostate cancer, this effect appeared to be largely driven by one trial of the drug sodium clodronate, and our results suggest that any potential benefit of zoledronic acid is small. We found no evidence that bisphosphonates improve survival in men with M0 disease.

The results are reliable and robust for men with M1 hormone-sensitive prostate cancer treated with docetaxel because, although based on three trials only, these results are derived from 93% of all men who were randomly assigned to treatment groups and 1271 deaths. Although additional results might become available in this setting, from both the GOUP 01/04 and the GENTAX^32^ trials, these results are unlikely to materially affect our findings. Importantly, however, in three of the included trials^7^, ^8^, ^10^ most of the men who were randomly assigned to treatment groups were newly diagnosed with metastatic disease. A few men had progressed after previous diagnoses of localised disease, and results for this specific subgroup were not reported. While we see no reason for why the observed benefit of docetaxel should not be generalisable, the only way to appropriately assess this, or any other remaining questions, is through the collection and re-analysis of individual participant data. Across the three trials, the number of reported grade 3–4 toxic effects increased with docetaxel, most commonly neutropenia. Overall, 16 deaths were attributed to docetaxel. Nevertheless, docetaxel combined with androgen deprivation therapy should be considered a new standard of care for men with metastatic disease starting on long-term androgen deprivation therapy for the first time who are fit to receive chemotherapy and willing to accept these risks. Future trials in this setting should also consider this as an appropriate control group.^48^

In men with non-metastatic disease, we found evidence that docetaxel improves failure-free survival; however, this conclusion is based on data from four trials including just over half of all men who were randomly assigned to treatment groups. Nevertheless, as the estimate of effect (HR 0·70) is in keeping with that for men with metastatic disease (HR 0·64) and the confidence interval is narrow, this finding provides a clear and early signal of potential benefit. For overall survival, however, the available data are less mature, such that the estimate of effect is based on half of all men who were randomly assigned to treatment groups and 340 deaths, and the confidence interval is wide. This meta-analysis will be important to update, to include mature results of unreported trials and long-term follow-up of those already reported, to reliably assess any effect of docetaxel on survival. We will need to collaborate with trial investigators to determine when these data are likely to emerge so that we can predict when a meta-analysis that includes a much larger proportion of the men randomised in this setting and provides sufficient power to detect moderate survival benefits will be feasible. Importantly, as a notable proportion of men will die from causes other than prostate cancer, any treatment effect on survival is likely to be diluted. Thus, we will also need to examine the effects of docetaxel on prostate cancer-specific survival, which will only be possible through our planned international individual participant data meta-analysis.

Despite the benefits of bisphosphonates with respect to skeletal-related events and bone pain,^49^, ^50^ the effect of bisphosphonates on survival in men with hormone-sensitive prostate cancer is less clear. In men with M1 disease, although based only on three trials, these results represent 87% of men who were randomly assigned to treatment groups and suggest a small potential survival benefit. However, this result is driven largely by the outcome of the PR05 trial,^12^ which showed a benefit of sodium clodronate. In view of the differences in mechanisms of action between clodronate and zoledronic acid, we planned analyses that considered trials of the two treatments separately. Moreover, as sodium clodronate is not commonly used in practice, our focus is on the findings relating to zoledronic acid. The four additional trials, which have yet to report survival, randomly assigned fewer than 400 patients in total and so will probably not alter the results. Moreover, the results to date suggest that any absolute benefit from zoledronic acid is likely to be small at best. In the non-metastatic setting, although based on only four of 17 trials, the analysis includes around 65% of randomly assigned men, and we found no evidence of a benefit of bisphosphonates on survival. Data from other identified trials might provide enough power to detect a small benefit, but our results at present suggest that even a small benefit of zoledronic acid is unlikely.

In both the metastatic and non-metastatic disease settings, we are aware of a number of limitations of a meta-analysis based on the reported trials of bisphosphonates, not least that many of the trials identified in the systematic review have not reported survival and so could not contribute to the meta-analysis. Crossover policies and actual treatment on progression varies between the included trials. For example, in the STAMPEDE trial,^8^ treatment was stopped at the time of progression, whereas in the CALGB 90202 trial,^30^ patients crossed over to receive zoledronic acid when evidence of biochemical failure was found. The potential effect of treatment crossover on overall survival is unclear. Therefore, an analysis of failure-free survival remains important; however, variations in definition meant that this was not possible from the reported data. The collection of individual participant data, or alternatively, provision or consistent reporting of results would enable us to better ascertain the role for bisphosphonates on other outcomes. The ongoing ICECaP initiative^51^ should help define the most appropriate intermediate outcomes in men with hormone-sensitive prostate cancer.

Rigorous systematic review methods helped us identify all relevant trials in the two treatment comparisons, irrespective of whether they were completed or reported. This approach allowed us to decide prospectively when we would be likely to have sufficient data and power to detect meaningful effects of docetaxel or bisphosphonates in combination with standard of care, at least in the M1 disease setting. Despite knowing that there would be fewer data and less power to assess the effects of both treatments in the non-metastatic disease setting than in the metastatic disease setting, we have been able to establish early signals of both benefit (docetaxel) and no benefit (bisphosphonates), consistency of results with those in metastatic disease, and whether new data are likely to change the results. By using an approach that is responsive to the emerging trial results and adaptive to potential future data, we have been able to achieve robust answers to specific therapeutic questions quickly and determine which meta-analyses will need updating in the future and which will require individual patient data for more reliable and detailed results.

In summary, for men with metastatic prostate cancer starting therapy for the first time, we found strong evidence to support the addition of docetaxel to androgen deprivation therapy as the new standard of care, and this combination should be offered to men who are fit to receive chemotherapy. More reliable evidence of the effect of docetaxel on overall survival and prostate cancer-specific survival is still needed in the M0 disease setting and will be achieved through our planned collaborative international meta-analysis of individual participant data. This project will also allow us to investigate whether effects vary by patient or tumour characteristics. We found no evidence that zoledronic acid improves survival in men with either metastatic or non-metastatic hormone-sensitive disease. Although additional trials are yet to be reported, the suggestion from our analyses is that any likely benefit of zoledronic acid will probably be small and not clinically meaningful.

**This online publication has been corrected. The corrected version first appeared at thelancet.com/oncology on February 2, 2016**

## Figures and Tables

**Table 1:** Characteristics of studies included in the systematic review and meta-analysis

	**Accrual period**	**Number of patients**	**Control**	**Treatment**	**Metastatic status**	**Median age (range)**	**Gleason score of 8–10 (%)**	**Performance status of 0–1 (%)**	**Median follow-up (survival)**	**Treatment on progression (control group only)**
**Docetaxel trials**										
GETUG-12^25^, ^26^	November, 2002–December, 2006	413	ADT (goserelin 10·8 mg every 3 months for 3 years)	ADT plus docetaxel (70 mg/m^2^ for four cycles) plus estramustine	M0	63 (46–77)	42%	Unknown	7 years, 6 months	Not reported
TAX 3501^27^	December, 2005–September, 2007	228	ADT (leuprolide 22·5 mg every 3 months for 18 months)	ADT plus docetaxel (75 mg/m^2^ every 3 weeks for six cycles)	M0	61·9*	52%	Unknown	3 years, 3 months	Not reported
RTOG 0521^28^	December, 2005–August, 2009	612	ADT (LHRH agonist plus oral anti-androgen plus RT)	ADT plus docetaxel (75 mg/m^2^ every 3 weeks for six cycles) plus prednisone	M0	66 (unknown)	84%	Unknown	6 years	Not reported
STAMPEDE (standard of care with or without docetaxel)^8^	September, 2005–March, 2013	1776	ADT (plus radiotherapy for M0 patients)	ADT plus docetaxel (75 mg/m^2^ every 3 weeks for six cycles) plus predisone	M0 and M1	65 (40–82)	70%	99%	3 years, 6 months	40% received docetaxel (49% received life-extending treatments)
STAMPEDE (standard of care plus zoledronic acid with or without docetaxel)^8^	September, 2005–March, 2013	1186	ADT (plus radiotherapy for M0 patients) plus zoledronic acid (4 mg every 3–4 weeks for 2 years)	ADT (plus radiotherapy for M0 patients) + zoledronic acid (4 mg for 3–4 weeks for 2 years) plus docetaxel (75 mg/m^2^ every 3 weeks for six cycles)	M0 and M1	66 (42–84)	71%	99%	3 years, 6 months	36% received docetaxel (45% received life-extending treatments)
GETUG-15^9^, ^10^	October, 2004–December, 2008	385	ADT (LHRH agonist or surgical castration or combined androgen blockade)	ADT plus docetaxel (75 mg/m^2^ every 3 weeks for up to nine cycles)	M1	63·5 (57–70)	56%	100%	6 years, 11 months	62% received docetaxel
CHAARTED^7^	July, 2006–November, 2012	790	ADT (LHRH agonist or LHRH antagonist) or surgical castration	ADT plus docetaxel (75 mg/m^2^ every 3 weeks for six cycles)	M1	64 (36–91)	61%	98%	2 years, 5 months	147 (51%) of 287 men received docetaxel (104 of 287 men received abiratarone or enzalutamide)
**Bisphosphonate trials**										
PRO4^12^	June, 1994–December, 1997	508	Local standard practice (radiotherapy or hormone therapy or both) plus placebo	Local standard practice plus clodronate (520 mg four times daily)	M0	69·5 (49–87)	Unknown	97%	12 years	Not reported
RADAR^21^	October, 2003–August, 2007	1071	ADT (leuprorelin 22·5 mg for either 6 months or 18 months)	ADT plus zoledronic acid (4 mg every 3 months for 18 months)	M0	68·8 (62·6–73·3)	35%	100%	7 years, 5 months	Secondary therapeutic intervention was needed in 78 men in the short-term androgen suppression group, and 61 men in the intermediate-term androgen suppression group; nature of treatment not reported
ZEUS^29^	June, 2004–August, 2007	1433	ADT	ADT plus zoledronic acid (4 mg every 3 months for up to 4 years)	M0	67 (44–87)	62%	100%	4 years, 9 months	Not reported
STAMPEDE (standard of care with or without zoledronic acid)^8^	September, 2005–March, 2013	1777	ADT (plus radiotherapy for M0 patients)	ADT (plus radiotherapy for M0 patients) plus zoledronic acid (4 mg every 3–4 weeks for 2 years)	M0 and M1	66 (41–82)	69%	99%	3 years, 7 months	40% received docetaxel (49% received life-extending treatments)
STAMPEDE (standard of care plus docetaxel with or without zoledronic acid)^8^	November, 2005–March, 2013	1185	ADT (plus radiotherapy for M0 patients) + docetaxel (75mg/m^2^/3wks/6cycles)	ADT (plus radiotherapy for M0 patients) plus docetaxel (75 mg/m^2^ every 3 weeks for six cycles) plus zoledronic acid (4 mg every 3–4 weeks for 2 years)	M0 and M1	66 (40–84)	73%	99%	3 years, 7 months	14% received further docetaxel (41% received life-extending treatments)
PRO5^12^	June, 1994–July, 1998	311	Local standard practice=radiotherapy or hormone therapy or both plus placebo	Local standard practice plus clodronate (520 mg four times daily)	M1	71 (47–88)	Unknown	94%	11 years, 6 months	55 men received radiotherapy; 40 men “changed hormone therapy”
CALGB 90202^30^	June, 2004–April, 2012	645	ADT=bilateral orchidectomies, GnRH agonist or GnRH antagonist (and zoledronic acid placebo)	ADT plus zoledronic acid (4 mg intravenous every 4 weeks)	M1	66·3 (60–73)	58%	97%	2 years	49% of the men in zoledronic acid group and 51% of men in the placebo group initiated open-label treatment with zoledronic acid

ADT=androgen deprivation therapy. LHRH=luteinising hormone-releasing hormone. PSA=prostate-specific antigen.

* This value is the mean (no SD was available)

**Table 2:** Characteristics of studies included in the systematic review that could not be included in the meta-analyses

	**Accrual dates**	**Number of patients**	**Metastatic status**	**Primary outcome**	**Secondary outcomes**	**Reason not included**
**ADT *vs* ADT + docetaxel**						
ARTIC AOM-03108^31^	June, 2003–November, 2009	254	M0	PSA progression-free survival	PSA response; duration of PSA response; time to clinical progression; overall survival; tolerability; quality of life	Reported results could not be used (safety 2010, progression-free survival* 2011, quality of life 2013)
GENTAX^32^	October, 2005–December, 2009	30	M0 and M1	Progression-free survival	Overall survival; toxicity; quality of life	Reported results could not be used (progression-free survival*)
SPCG-13^33^	May, 2007–November, 2004	378	M0	PSA progression	PSA doubling time; quality of life; safety; metastasis-free survival; overall survival	Reported results could not be used (safety)
TAX 3503^34^	July, 2007–September, 2012	400	M0	Progression-free survival	Overall survival; cancer-specific survival; adverse events	Reported results could not be used (safety)
CAN-NCIC-PR12 (NCT00651326)	March, 2008–January, 2011	48	M0	Disease-free survival	Overall survival; time to biochemical disease progression; time to local or distant disease progression; time to next anti-cancer therapy; progression-free survival; degree of PSA suppression before radiotherapy; quality of life; adverse events	No results reported yet
QRT-SOGUG^35^	December, 2008–September, 2012	134	M0	PSA relapse	Unclear	Reported results could not be used (toxicity)
05-043 (NCT00116142)	June, 2005–August, 2015	350	M0	Overall survival	PSA doubling time; PSA failure; cancer-specific survival	No results reported yet
GOUP-01/04 (NCT00796458)	April, 2005–ongoing	200	M1	2-year progression-free survival	Overall survival; time to treatment failure; toxicity; PSA response rate; disease response rate; PSA normalisation; quality of life; control of bone pain; change in chromogranin A concentration; cost analysis	Ongoing
**ADT *vs* ADT + bisphosphonates**						
Smith 2005^36^	September, 1999–March, 2003	544	M0	Bone metastasis-free survival and overall survival	Time to first skeletal-related events; quality of life; pain	Reported results could not be used (overall survival*, time to first bone metastasis)
Ryan 2007^37^	January, 2000–December, 2002	42	M0 + M1	BMD	Urinary NTX concentration and serum BAP concentration	Reported results could not be used (bone mineral density, urinary NTX, serum BAP)
Smith 2003^38^	February, 2000–November, 2000	106	M0	LS BMD	Other bone mineral density	Reported results could not be used (bone mineral density)
Israeli^39^	February, 2003–May, 2005	222	M0	LS BMD	TH bone mineral density; serum NTX; serum BSAP	Reported results could not be used (LS bone mineral density, TH bone mineral density, serum NTX)
Ryan 2006^40^	April, 2003–March, 2004	120	M0	FN/LS BMD	Serum BSAP; urine NTX; TH BMD	Reported results could not be used (bone mineral density, urinary NTX, serum BSAP)
Zenith (NCT00063609)	April, 2003–April, 2005	200	M0	LS BMD	TH BMD; markers of bone turnover	No results reported yet
Rao^41^	June, 2003–May, 2004	50	M0	BMD	Urinary DPD	Reported results could not be used (BMD)
HOG GU02-41^42^	December, 2003–August, 2005	63	M1	Skeletal-related events	Time to castrate-resistant prostate cancer; markers of bone turnover	Reported results could not be used (skeletal-related events, castrate-resistant prostate cancer, serological progression, prostate-specific antigen nadir, adverse events, urine DPD, urine NTX, serum BAP)
Bhoopalam^43^	December, 2003–May, 2006	93	M0	LS bone mineral density	NA	Reported results could not be used (bone mineral density)
Casey^44^	Unclear	200	M0	LS bone mineral density	FN/TH BMD; change in height; safety	Reported results could not be used (bone mineral density)
Yedavelli^45^	Unclear	42	M0	Skeletal-related events	Bone mineral density	Reported results could not be used (bone mineral density)
Rodrigues^46^	Unclear	94	M0	Bone mineral density	NA	Reported results could not be used (bone mineral density)
CEGOG (NCT00294437)	December, 2003–November, 2007	376	M0	Time to bone metastasis	Pain; time to first bone pain; skeletal-related events; serum PSA; safety	No results reported yet
Ueno^47^	July, 2006–June, 2011	60	M1	PSA progression-free survival	Skeletal-related events; bone pain; markers of bone turnover	Reported results could not be used (PSA and progression-free survival,* skeletal-related events, bone pain)
KYUHTRIGU0705 (NCT00685646)	May, 2008–December, 2013	227	M1	Time to treatment failure	Time to first skeletal-related event; overall survival; extent of disease; pain	No results reported yet
NU-02U1 (NCT00058188)	March, 2003–September, 2015	70	M0	Bone mineral density	LS bone mineral density	No results reported yet

ADT=androgen deprivation therapy. NA=non-applicable. PSA=prostate-specific antigen. NTX=N-terminal telopeptide. BAP=bone alkaline phosphatase. LS BMD=lumbar spine bone mineral density. FN/LS BMD=femoral neck/lumbar spine bone mineral density. TH BMD=total hip bone mineral density. BSAP=bone-specific alkaline phosphatase. CRPC=castrate-resistant prostate cancer. DPD=deoxypridinoline.

* Data reported not usable.

**Table 3:** Assessment of risk of bias

	**Adequate sequence generation**	**Allocation concealment**	**Masking**	**Incomplete outcome data addressed**	**Free of selective reporting**
TAX 3501^27^	Randomisation with stratification factors reported	Randomised	NA	All randomised patients included in the analyses	Yes, although survival not reported, data not mature
CHAARTED^7^	Randomisation with stratification factors reported	Centrally randomised	NA	All randomised patients included in the analyses	Yes, all outcomes of interest are reported
GETUG-12^25^, ^26^	Randomisation with stratification factors reported	Centrally randomised	NA	All randomised patients included in the analyses	Yes, outcomes of interest are reported, although survival data reported are not yet mature
STAMPEDE^8^	Used a method of minimisation over a number of clinically important stratification factors with an additional random element	Central telephone randomisation	NA	All randomised patients included in the analyses	Yes, outcomes of interest are reported
RTOG 0521^28^	Randomisation with stratification factors reported	Centrally randomised	NA	45 ineligible patients (3% of the total) were excluded from analyses; not clear if balanced by treatment group	Yes, outcomes of interest are reported
GETUG-15^9^, ^10^	Minimisation method with stratification factors reported	Centrally randomised	NA	All randomised patients included in the analyses	Yes, outcomes of interest are reported
CALGB 90202^30^	Randomised block design with stratification factors	Central online registration and randomisations	Double-blind or placebo-controlled	All randomised patients are included in the efficacy analyses	Reports survival, but not failure-free survival as defined in the meta-analysis
RADAR^21^	Minimisation with a random element and stratification factors	Central trials office computer based randomisation	Open label; the endpoints committee were unaware of patient identity or treatment assignment; treatment was not masked to the investigators, patients, or trial statistician	All randomised patients are included in the efficacy analyses	Reports survival, but not failure-free survival as defined in the meta-analysis
ZEUS^29^	Minimisation method described by Pocock^53^ with stratification factors	Central randomisation by fax	Open label	40 patients (3% of total randomised) excluded from analyses; seven patients were ineligible; 27 patients withdrew consent; six patients were lost to follow-up; exclusions are balanced by group	Reports survival, but not failure-free survival as defined in the meta-analysis
PR04^12^	Minimisation method over five stratification factors	Central randomisation	Double blind; placebo-controlled; clinicians assessing cause of death were blinded to treatment allocation	In the primary analysis, no randomised patients were excluded from the analyses; in the analysis with long-term follow-up, 37 patients were excluded as they had not been flagged with the NHS Information Centre	Reports survival, but not failure-free survival as defined in the meta-analysis
PR05^12^	Minimisation method over four stratification factors	Central randomisation	Double blind; placebo controlled	In the primary analysis, no randomised patients were excluded from the analyses; in the analysis with long-term follow-up, 33 patients were excluded as they had not been flagged with the NHS Information Centre	Reports survival, but not failure-free survival as defined in the meta-analysis
STAMPEDE^8^	Used a method of minimisation over a number of clinically important stratification factors with an additional random element	Central telephone randomisation	Open label	All randomised patients included in the analyses	Yes, outcomes of interest are reported, including survival and failure-free survival

NA=non-applicable. NHS=National Health Service.
