# Correction to *Lancet Oncol* 2016; 17: 248, 252

**DOI:** 10.1016/S1470-2045(16)00020-6

**Published:** 2016-02

*Vale CL, Burdett S, Rydzewska LHM, et al. Addition of docetaxel or bisphosphonates to standard of care in men with localised or metastatic, hormone-sensitive prostate cancer: a systematic review and meta-analyses of aggregate data.* Lancet Oncol *2016; **17:** 243–56—*In the Results section, the third sentence of the second paragraph should read “In the three remaining trials,^7,8,10^ men aged 36–91 years (median 63–66 years) with a good performance status received androgen deprivation therapy-based treatments (standard of care) with or without docetaxel”. In figure 3, the text under parts A and C should have read “Favours SOC + bisphosphonate”, and the text under parts B and D should have read “Favours SOC + zoledronic acid”. These corrections have been made to the online version as of Feb 2, 2016, and the printed version is correct.

