# Supplementary material for: Addition of docetaxel or bisphosphonates to standard of care in men with localised or metastatic, hormone-sensitive prostate cancer: a systematic review and meta-analyses of aggregate data
Source: Lancet Oncol. 2016 Feb;17(2):243–56. doi: 10.1016/S1470-2045(15)00489-1 (PMC4737894; doi:10.1016/S1470-2045(15)00489-1)
Supplement: Supplementary appendix [file mmc1.pdf]

THE LANCET Oncology

Supplementary appendix

This appendix formed part of the original submission and has been peer reviewed. We post it as supplied by the authors.

Supplement to: Vale CL, Burdett S, Rydzewska LHM, et al, for the STOpCaP Steering Group. Addition of docetaxel or bisphosphonates to standard of care in men with localised or metastatic, hormone-sensitive prostate cancer: a systematic review and meta-analyses of aggregate data. *Lancet Oncol* 2015; published online Dec 21. http://dx.doi.org/10.1016/S1470-2045(15)00489-1.

Web Appendix

Search Strategies

MEDLINE Search strategy

Cochrane Highly Sensitive Search Strategy for identifying RCTs in MEDLINE: sensitivity and precision maximising version(14); Ovid format

RCT filter MEDLINE
1. "randomi*ed controlled trial".pt.
2. controlled clinical trial.pt.
3. "randomi*ed".ab.
4. placebo.ab.
5. clinical trials as topic.sh.
6. randomly.ab.
7. trial.ti.
8. 1 or 2 or 3 or 4 or 5 or 6 or 7 or 8
9. exp animals/ not humans.sh.
10. 8 not 9

AND
Terms specific to prostate cancer:

11. exp Prostatic Neoplasms/
12. (prostat$ adj3 adeno$).mp.
13. (prostat$ adj3 malignan$).mp.
14. (prostat$ adj3 canc$).mp.
15. (prostat$ adj3 carcinoma$).mp.
16. (prostat$ adj3 tumo?r$).mp.
17. (prostat$ adj3 neoplas$).mp.
18. 11 or 12 or 13 or 14 or 15 or 16 or 17

AND
Terms specific to Androgen deprivation Therapy (ADT)

19. ((androgen$ or hormon$) adj3 (ablat$ or block$ or withdraw$ or depriv$ or suppress$)).mp.
20. exp Antineoplastic Agents, Hormonal/tu [Therapeutic Use]
21. exp Androgen Antagonists/
22. (luteinizing hormone releasing hormone or LHRH).mp.
23. Orchiectomy.mp.
24. 19 or 20 or 21 or 22 or 23

AND
Terms specific to drug therapy:

25. exp Drug Therapy/
26. (multimodal$ or adjuvant or adjunct$).mp.
27. (together or plus or concurrent or combin$ or add$ or conjuct$).tw.
28. 25 or 26 or 27

AND
Terms specific to chemotherapy

29. ((chemotherapy or antineoplastic or anticancer) adj3 (agent$).tw.
30. exp chemotherapy adjuvant/
31. exp Antineoplastic agents/
32. docetaxel.tw.
33. taxotere.tw.
34. 29 or 30 or 31 or 32 or 33

AND
Terms specific to bisphosphonates:

35. exp diphosphonates/
36. exp bisphosphonates/
37. (bisphosphonate$ or disphosphonat$).af.
38. zoledron$.af.
39. zometa.af.
40. aclasta.af.

41. 35 or 36 or 37 or 38 or 39 or 40

COMBINE ALL

42. 10 and 18 and 24 and 28
43. 34 or 41
44. 42 and 43

/ means all subheadings were selected
pt = publication type
mp = free text search for a term
tw term in a title/ abstract
af term in all fields

EMBASE Search strategy

Best Optimisation of Sensitivity and Specificity Search(15); Ovid format

RCT filter EMBASE
1. randomi:.tw.
2. placebo:.mp.
3. double-blind:.tw.
4. 1 or 2 or 3

AND
Terms specific to prostate cancer:
5. exp Prostatic Neoplasms/
6. (prostat$ adj3 adeno$).mp.
7. (prostat$ adj3 malignan$).mp.
8. (prostat$ adj3 canc$).mp.
9. (prostat$ adj3 carcinoma$).mp.
10. (prostat$ adj3 tumo?r$).mp.
11. (prostat$ adj3 neoplas$).mp.
12. 5 or 6 or 7 or 8 or 9 or 10 or 11

AND
Terms specific to Androgen deprivation Therapy (ADT)
13. ((androgen$ or hormon$) adj3 (ablat$ or block$ or withdraw$ or depriv$ or suppress$)).mp.
14. exp Antineoplastic Agents, Hormonal/tu [Therapeutic Use]
15. exp Androgen Antagonists/
16. (luteinizing hormone releasing hormone or LHRH).mp.
17. Orchi$ectomy.mp.
18. 13 or 14 or 15 or 16 or 17

AND
Terms specific to drug therapy:
19. exp Drug Therapy/
20. (multimodal$ or adjuvant or adjunct$).mp.
21. (together or plus or concurrent or combin$ or add$ or conjuct$).tw.
22. 19 or 20 or 21

AND
Terms specific to chemotherapy
23. ((chemotherapy or antineoplastic or anticancer) adj3 (agent$).tw.
24. exp chemotherapy adjuvant/
25. exp Antineoplastic agents/
26. docetaxel.tw.
27. taxotere.tw.
28. 23 or 24 or 25 or 26 or 27

AND
Terms specific to bisphosphonates:
29. exp diphosphonates/
30. exp bisphosphonates/
31. (bisphosphonate$ or disphosphonat$).af.
32. zoledron$.af.
33. zometa.af.
34. aclasta.af.
35. 29 or 30 or 31 or 32 or 33 or 34

COMBINE ALL
36. 4 and 12 and 18 and 22
37. 28 or 35
38. 36 and 37

LILACS Search strategy

Highly Sensitive Search Strategy(16); Lilacs format

ENTERED AS:
(Tw estud$ OR Tw clinic$ OR AB grupo$ OR CT COMPARATIVE STUDY OR Tw placebo$ OR Tw random$ OR Ti compara$ OR Ti tratamiento OR Tw control$ OR MH/dt) AND NOT ((CT ANIMALS FEMALE OR CT ANIMALS MALE OR CT CATS OR CT CATTLE OR CT CHICK EMBRYO OR CT DOGS OR CT GUINEA PIGS OR CT IN VITRO OR CT MICE OR CT RABBITS OR CT RATS) OR (MH Prevalence OR MH Practice Guidelines OR MH Diagnosis, Differential OR MH Cross-Sectional Studies OR MH predictive value of tests) OR (Ti clinical AND case OR Ti updat$ OR Ti Epidemiol$ OR Ti clinical$ AND case$ OR Ti caso AND clinico OR Ti review OR Ti diagno$ AND treatment OR Ti descrip$ OR Ti consenso OR Ti caso$ AND control$ OR Ti analisis AND critico) OR (AB retrospectiv$ and stud$ OR AB estudio AND retrospectivo OR AB revis$ AND ficha$ OR AB revision AND bibliograf$ OR AB estud$ AND descript$ OR AB presenta AND caso OR AB describe AND caso OR AB serie AND clinica OR AB puesta AND al AND dia OR AB tratamiento AND diagnostic$ AND revis$ OR AB experien$ AND caso$ OR AB analisis AND critico) OR (PT case reports OR PT review) AND NOT (Tw estud$ OR AB grupo$ OR Tw control$ OR Tw random$)) [Words]
And
(Mh prostatic neoplasms/) or (tw prostat$ AND (Tw carcinoma$ or Tw canc$ or Tw tumo$ or Tw neoplas$)) [Words]
And
(Mh Drug therapy/) or (Tw chemotherapy) [Words]

CENTRAL Search strategy

RCT filter not required for CENTRAL (all RCTs)

Terms specific to prostate cancer:
MeSH descriptor: [Prostatic Neoplasms] explode all trees
(prostat* near adeno*):ti,ab,kw
(prostat* near malignan*):ti,ab,kw
(prostat* near canc*):ti,ab,kw
(prostat* near carcinoma*):ti,ab,kw
(prostat* near tumo?r*):ti,ab,kw
(prostat* near neoplas*):ti,ab,kw
#1 or #2 or #3 or #4 or #5 or #6 or #7
Terms specific to Androgen deprivation Therapy (ADT)
((androgen* or hormon*) near (ablat* or block* or withdraw* or depriv* or suppress*)):ti,ab,kw
MeSH descriptor: [Antineoplastic Agents, Hormonal] explode all trees
MeSH descriptor: [Androgen Antagonists] explode all trees
(luteini?ing hormone releasing hormone or LHRH):ti,ab,kw
Orchi*ectomy:ti,ab,kw
#9 or #10 or #11 or #12 or #13
Terms specific to drug therapy:
MeSH descriptor: [Drug Therapy] explode all trees
Terms specific to chemotherapy
((chemotherapy or antineoplastic or anticaner) near (agent*)):ti,ab,kw
MeSH descriptor: [Chemotherapy, Adjuvant] explode all trees
MeSH descriptor: [Antineoplastic Agents] explode all trees
MeSH descriptor: [Antineoplastic Combined Chemotherapy Protocols] explode all trees
#15 or #16 or #17 or #18 #19
COMBINE ALL
#8 and #14 and #20

STOpCaP Meta-analysis Collaborative Group

Project Management Group
Sarah Burdett, Claire Vale, Larysa HM Rydzewska, Jayne F Tierney, Matthew Sydes, David Fisher - MRC Clinical Trials Unit at UCL, London, UK

International Advisory Group
Laurence Albiges, Institut Gustave Roussy, University of Paris Sud, Villejuif, France
Noel Clarke, The Christie NHS Foundation Trust, Manchester University, Manchester, UK
Karim Fizazi, Institut Gustave Roussy, University of Paris Sud, Villejuif, France
Gwenaelle Gravis, Institut Paoli Calmettes, Marseille, France
Nicholas James, University of Warwick and Queen Elizabeth Hospital, Birmingham, UK
Malcolm Mason, Cardiff University School of Medicine, UK
Mahesh Parmar, MRC Clinical Trials Unit at UCL, London, UK
Christopher Sweeney, Dana-Farber Cancer Institute, Boston, MA, USA
Bertrand Tombal, Cliniques Universitaires St Luc, Brussels, Belgium
